# Supplementary material for: Cerianthus lloydii (Ceriantharia: Anthozoa: Cnidaria): New Status and New Perspectives
Source: Biology (Basel). 2023 Aug 24;12(9):1167. doi: 10.3390/biology12091167 (PMC10525267; doi:10.3390/biology12091167)

Supplementary Table S1. Museum catalog numbers, GenBank accession numbers and geographical localities of specimens used in the study.

ID	Species	Region	locality verbal	Latitude	Longitude	Depth, m	NCBI accession number 18S	COI
IORAS CNI00025	*Arachnanthus sarsi* .	Northern East Atlantic	UK, Rathlin Island*	55.29	-06.22	10-12	OR127166	OR126090
USNM 1467536	*Botrucnidifer* sp.	Central North Pacific	Musician Seamounts; Beethoven Ridge; EX1708; DIVE03; SPEC02BIO	26.22	-159.15	2472.6	OR127168	OR126092
IORAS CNI00019	*Botrucnidifer* sp.	North Pacific	Bering Sea, Commander Basin	55.51	167.30	3545	OR127175	OR126099
USNM 1457395	Ceriantharia *Gen.nov. sp.nov.*	Central North Pacific	North Pacific Johnston Atoll; EX1706; DIVE12; SPEC02BIO	14.53	-169.92	2307.34	OR127167	OR126091
UF3748	*Ceriantheopsis americana*	Northern West Atlantic	USA, Florida, Seahorse key	29	-83	intertidal	OR127158	OR126081
IORAS CNI00009	*Ceriantheopsis* sp.1	South Indian Ocean	South Africa; Knysna; Thesens Island*	-34.05	23.05	5	OR127156	OR126080
IORAS CNI00006	*Pachycerianthus solitarius*	Mediterranean Sea	NW Mediterranean Sea, 3PP Cave*	43.16	5.60	15	—	OR126083
IORAS CNI00010	*Synarachactis lloydii*	Northern East Atlantic	UK, Plymouth, Firestone Bay, Eastern Kings*	50.36*	-4.16*	6-8	OR127157	OR126079
IORAS CNI00020	*Synarachactis lloydii*	Northern East Atlantic	UK, Plymouth, Firestone Bay, Eastern Kings*	50.36*	-4.16*	6	OR127161	OR126085
IORAS CNI00021-1	*Synarachactis lloydii*	Boreal Arctic	Barents Sea, Teriberka*	69.19*	35.15*	20-22	OR127162	OR126086
IORAS CNI00021-2	*Synarachactis lloydii*	Boreal Arctic	Barents Sea, Teriberka*	69.19*	35.15*	20-22	OR127164	OR126088
IORAS CNI00021-3	*Synarachactis lloydii*	Boreal Arctic	Barents Sea, Teriberka*	69.19*	35.15*	20-22	OR127165	OR126089
IORAS CNI00027	*Synarachactis lloydii*	Northern East Atlantic	Norwegian sea; Skjerstad Fjord	67.24	14.80	544	OR127174	OR126098

ID	Species	Region	locality verbal	Latitude	Longitude	Depth, m	NCBI accession number 18S	COI
IORAS CNI00028	*Synarachactis lloydii*	Black Sea	North East Black sea, off Divnomorskoe*	44.44*	38.03*	88	OR127153	OR126075
IORAS CNI00030	*Synarachactis lloydii*	Black sea	North East Black sea, off Divnomorskoe*	44.44*	38.03*	88	OR127154	OR126076
IORAS CNI00031	*Synarachactis lloydii*	Black Sea	North Est Black Sea, Kalamita Bay *	44.96*	33.10*	91	OR127155	OR126077
IORAS CNI00032	*Synarachactis lloydii*	Black Sea	North East Black sea, off Divnomorskoe*	44.44*	38.03*	108	—	OR126078
IORAS CNI00023	*Synarachactis roulei*	Arctic	White Sea, Kandalaksha Gulf, off Cape Kindo*	66.538	33.198	18-24	OR127163	OR126087
IORAS CNI00026	*Synarachactis roulei*	Arctic	Svalbard, Sassenfjorden, Diabasodden*	78.36	16.13	5	OR127159	OR126082
IORAS CNI00022	*Synarachactis roulei*	North Pacific	Bering Sea, Avachiskaya Guba	52.96*	158.59*	25	OR127160	OR126084
IORAS CNI00035	*Synarachactis roulei*	Arctic	Laptev Sea; Severnaya Zemlya; Bolshevik Il.; Mikoyan Bay*	79.27*	102.09*	4-7	OR127169	OR126093
IORAS CNI00036	*Synarachactis roulei*	Arctic	Laptev Sea; Severnaya Zemlya; Bolshevik Il.; Mikoyan Bay*	79.27*	102.09*	4-7	OR127170	OR126094
IORAS CNI00037	*Synarachactis roulei*	Arctic	Laptev Sea; Severnaya Zemlya; Bolshevik Il.; Mikoyan Bay*	79.27*	102.09*	4-7	OR127171	OR126095
IORAS CNI00038	*Synarachactis roulei*	Arctic	Laptev Sea; Severnaya Zemlya; Bolshevik Il.; Akhmatov Bay*	79.07*	103.02*	4-7	OR127172	OR126096
IORAS CNI00039	*Synarachactis roulei*	Arctic	Laptev Sea; Severnaya Zemlya; Bolshevik Il.; Akhmatov Bay*	79.07*	103.02*	4-7	OR127173	OR126097
IORAS CNI00033	*Synarachactis roulei*	Arctic	Vilkitsky Strait	78.27	105.1	100-105	OR127151	OR126073

ID	Species	Region	locality verbal	Latitude	Longitude	Depth, m	NCBI accession number	
							18S	COI
IORAS CNI00034	*Synarachactis roulei*	Arctic	Vilkitsky Strait	77.86	105.25	218-225	OR127152	OR126074

*Approximated from verbal

SupplementaryTable S2. Data obtained from public databases

	Species	Genbank accession numbers or BOLD sequence ID	
		**18S**	**COI**
**1**	*Ceriantheomorphe brasiliensis*	JF915195.1	AB859823.1
**2**	*Isarachnanthus maderensis*	JX128313.1	AB859825.1
**3**	*Pachycerianthus magnus*	AB859841.1	AB859828.1
**4**	*Pachycerianthus schlenzae* SS63	AB859840.1	AB859829.1
**5**	*Ceriantheopsis americana*	no data	AB859839.1
**6**	*Ceriantheopsis americana*	no data	JX023262.1
**7**	*Leiopathes* sp SCF 2009	FJ389898.1	FJ381663.1
**8**	*Botruanthus benedeni* CNI00004	MN582113.1	MN543659.1
**9**	*Botruanthus benedeni* Co3009	MN582115.1	MN543661.1
**10**	*Cerianthus cf. mortenseni* Co3002	MN582119.1	MN543664.1
**11**	*Cerianthus* sp. RS03	MN582120.1	MN543665.1
**12**	*Cerianthus membranaceus* Co2834	MN582122.1	MN543667.1
**13**	*Cerianthus membranaceus* Co3007	MN582126.1	MN543671.1
**14**	*Cerianthus membranaceus* MPIT01	MN582127.1	MN543672.1
**15**	*Botrucnidifer* sp. Co2832	MN582118.1	MN543674.1
**16**	*Isarachnanthus maderensis* ANT141	MN582129.1	MN543675.1
**17**	*Isarachnanthus maderensis* Co3008	MN582131.1	MN543677.1
**18**	*Pachycerianthus dohrni* Co2997	MN582138.1	MN543684.1
**19**	*Pachycerianthus dohrni* SZNCM2	MN582140.1	MN543686.1
**20**	*Pachycerianthus fimbriatus* Co2865	MN582141.1	MN543687.1

	Species	Genbank accession numbers or BOLD sequence ID	
		**18S**	**COI**
**21**	*Pachycerianthus multiplicatus* ANT003	MN582144.1	MN543690.1
**22**	*Pachycerianthus multiplicatus* Co3003	MN582146.1	MN543692.1
**23**	*Pachycerianthus torreyi* Co2998	MN582148.1	MN543694.1
**24**	*Pachycerianthus torreyi* Co3000	MN582150.1	MN543696.1
**25**	*Isarachnanthus* sp. UF:IZ:13274-Cnidaria	no data	MW278396.1
**26**	*Cerianthus lloydi* Anthozoa_1110V	no data	MG934859.1
**27**	Cerianthidae HCHAR585-19	no data	HCHAR585-19.COI-5P
**28**	Cerianthidae HCHAR590-19	no data	HCHAR590-19.COI-5P
**29**	*Pachycerianthus borealis* KHA717-14	no data	KHA717-14.COI-5P
**30**	*Pachycerianthus borealis* KHA718-14	no data	KHA718-14.COI-5P
**31**	*Pachycerianthus borealis* KHA719-14	no data	KHA719-14.COI-5P
**32**	*Pachycerianthus borealis* KHA720-14	no data	KHA720-14.COI-5P
**33**	*Pachycerianthus borealis* KHA721-14	no data	KHA721-14.COI-5P
**34**	*Cerianthus* sp. LABBI097-09	no data	LABBI097-09.COI-5P

SupplementaryTable S3 Estimates of Evolutionary Divergence over Sequence Pairs between and within (blue diagonal) clades calculated from 18S (grey) and COI (orange) sequences

18S \ COI	*Leiopathes*	**A3** *Synarachnactis roulei*	**A3** *S. lloydii*	**D Arachnactidae**	**NA gen_nov**	**A2** *Botrucnidifer*	**B** *Botruanthus -Cerianthus*	**C** *Pachycerianthus*	**A1** ***Ceriantheopsis_sp.***
Leiopathes	n/c / n/c	0,27	0,27	0,28	0,27	0,25	0,25	0,25	0,24
**A3** ***S. roulei***	0,07	0,002 / 0,003	0,01	0,17	0,15	0,12	0,14	0,17	0,14
**A3** ***S. lloydii***	0,07	0,01	0 / 0,004	0,17	0,15	0,12	0,14	0,16	0,14
**D Arachnactidae**	0,06	0,05	0,04	0,008 / 0,045	0,19	0,18	0,15	0,14	0,17
**gen_nov**	0,04	0,06	0,06	0,04	n/c / n/c	0,16	0,16	0,17	0,16
**A2** ***Botrucnidifer***	0,06	0,05	0,05	0,03	0,05	0,016 / 0,074	0,15	0,15	0,14
**B** ***Botruanthus - Cerianthus***	0,05	0,04	0,04	0,02	0,04	0,03	0,012 / 0,046	0,13	0,14
**C** ***Pachycerianthus***	0,05	0,05	0,04	0,01	0,04	0,03	0,01	0,002 / 0,038	0,15
**A1** ***Ceriantheopsis_sp.***	0,06	0,05	0,05	0,04	0,05	0,03	0,04	0,03	n/c / 0,003

SupplementaryTable S4. Estimates of Evolutionary Divergence over Sequence Pairs between and within clades calculated from COI sequences of Clade A representatives

	Synarachanctis roulei	*S.lloydii*	***Synarachnactis* indet. («*P.borealis*»)**	**Outgroup (*Botrucnidifer* + *Ceriantheopsis*)**
S. roulei	0,001			
S. lloydii	0,01	0,001		
***Synarachnanthus* indet. («*Pachycerianthus borealis*»)**	0,01	0,02	0	
**Outgroup (*Botrucnidifer* + *Ceriantheopsis*)**	0,13	0,13	0,13	0,137

Supplementary Figure S1. BI phylogenetic reconstruction based on concatenated alignment of 18S and COI loci. Posterior probabilities are shown. Model selected: GTR+I+G:18S, SYM+G: COI (1 position), HKY+G: COI (2 position), HKY+I+G: COI (3 position).

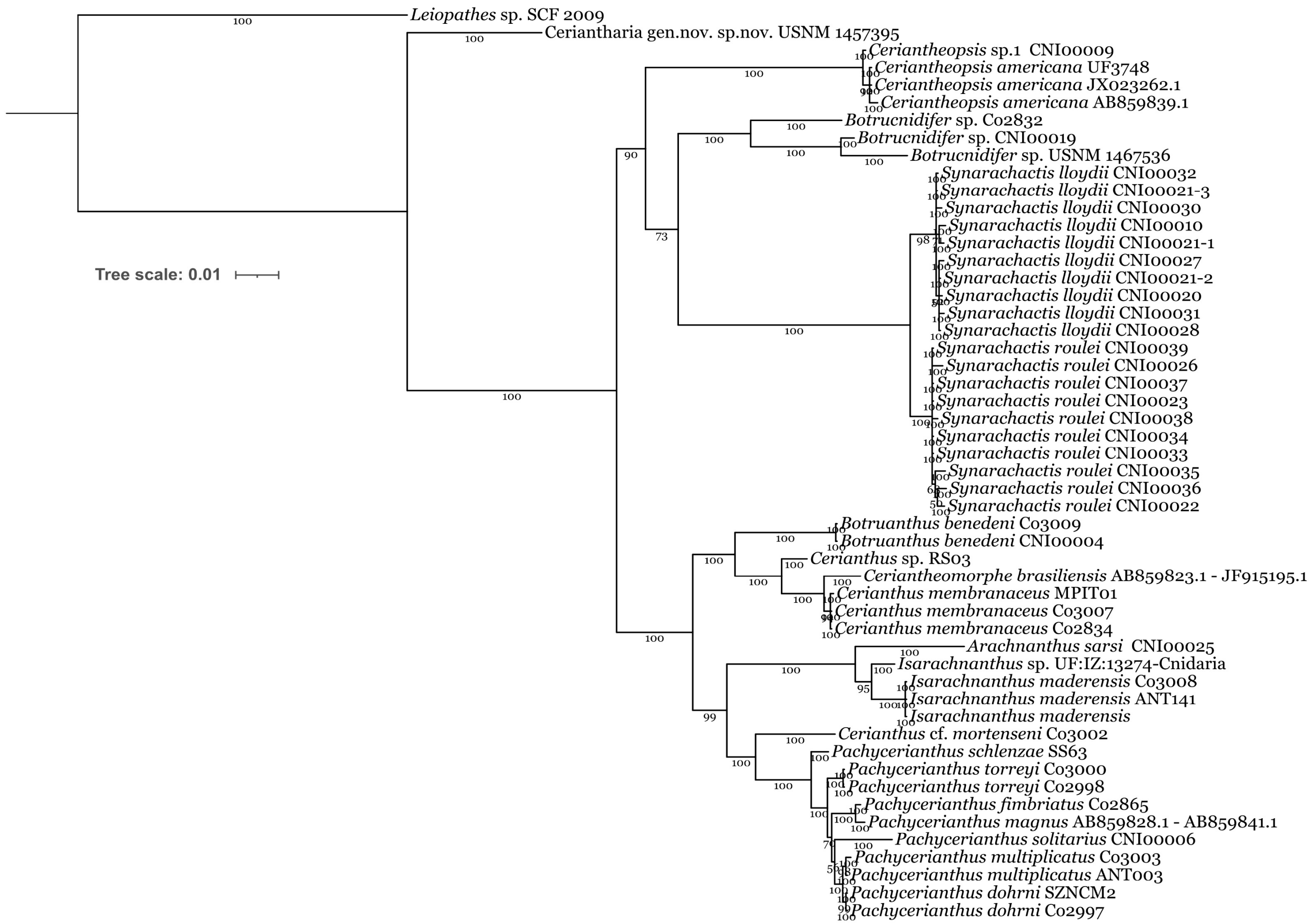

Supplementary Figure S2. COI ML phylogenetic reconstruction. The SH-aLRT support (%) / ultrafast bootstrap support (%) are shown. Model selected: TIM2+F+I+G4: COI (1 position), TIM2e+G4: COI (2 position), HKY+F+G4: COI (3 position).

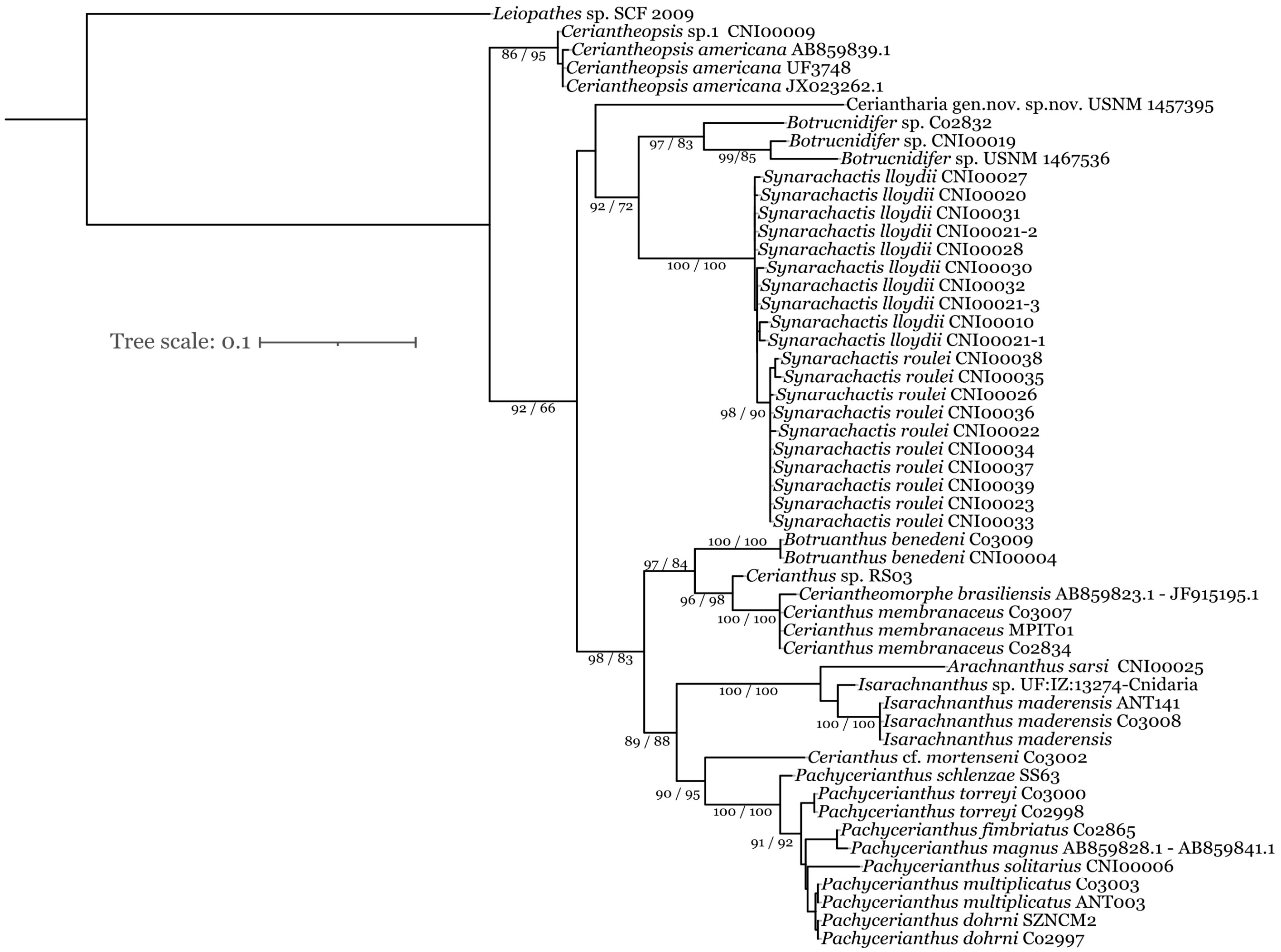

Supplementary Figure S3. COI BI phylogenetic reconstruction. Posterior probabilities are shown. Model selected: HKY+I+G: COI (1 position), K80+G: COI (2 position), HKY+G: COI (3 position).

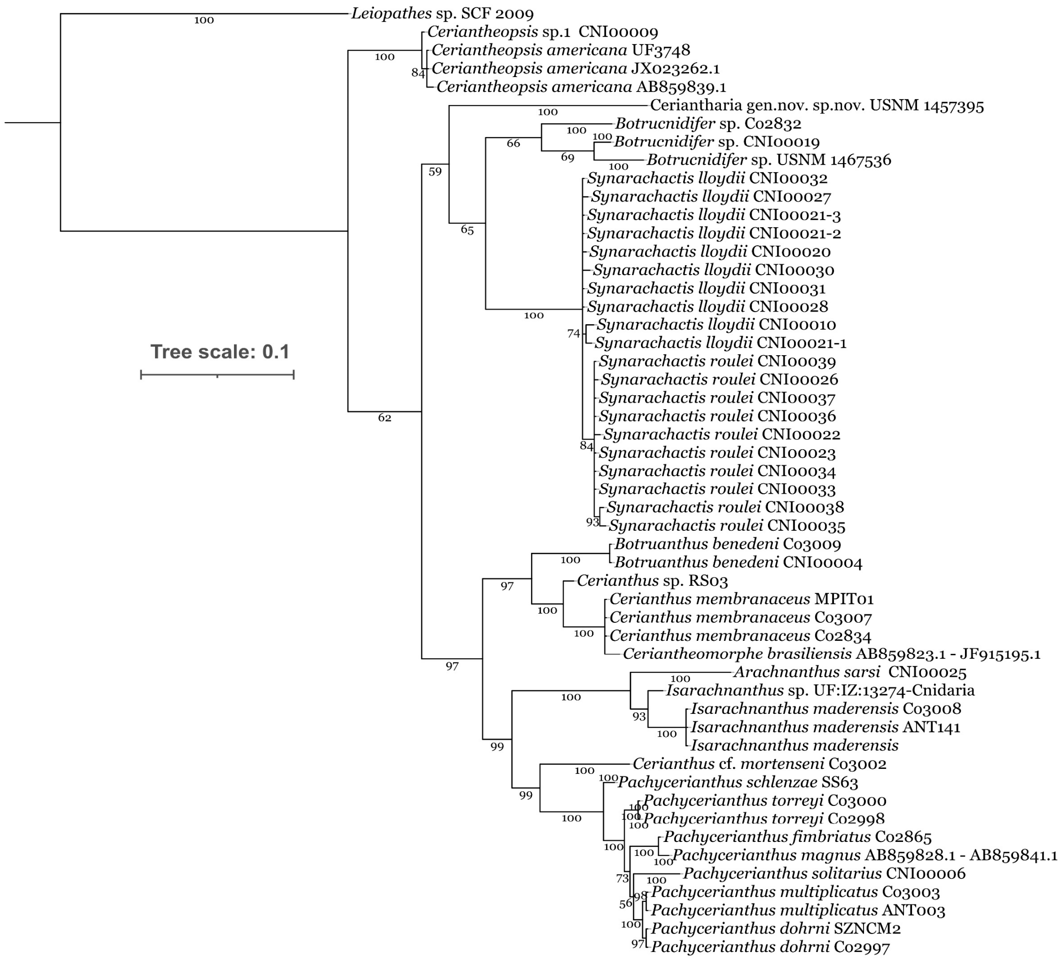

Supplementary Figure S4. 18S ML phylogenetic reconstruction. The SH-aLRT support (%) / ultrafast bootstrap support (%) are shown. Model selected: TIM2e+R2.

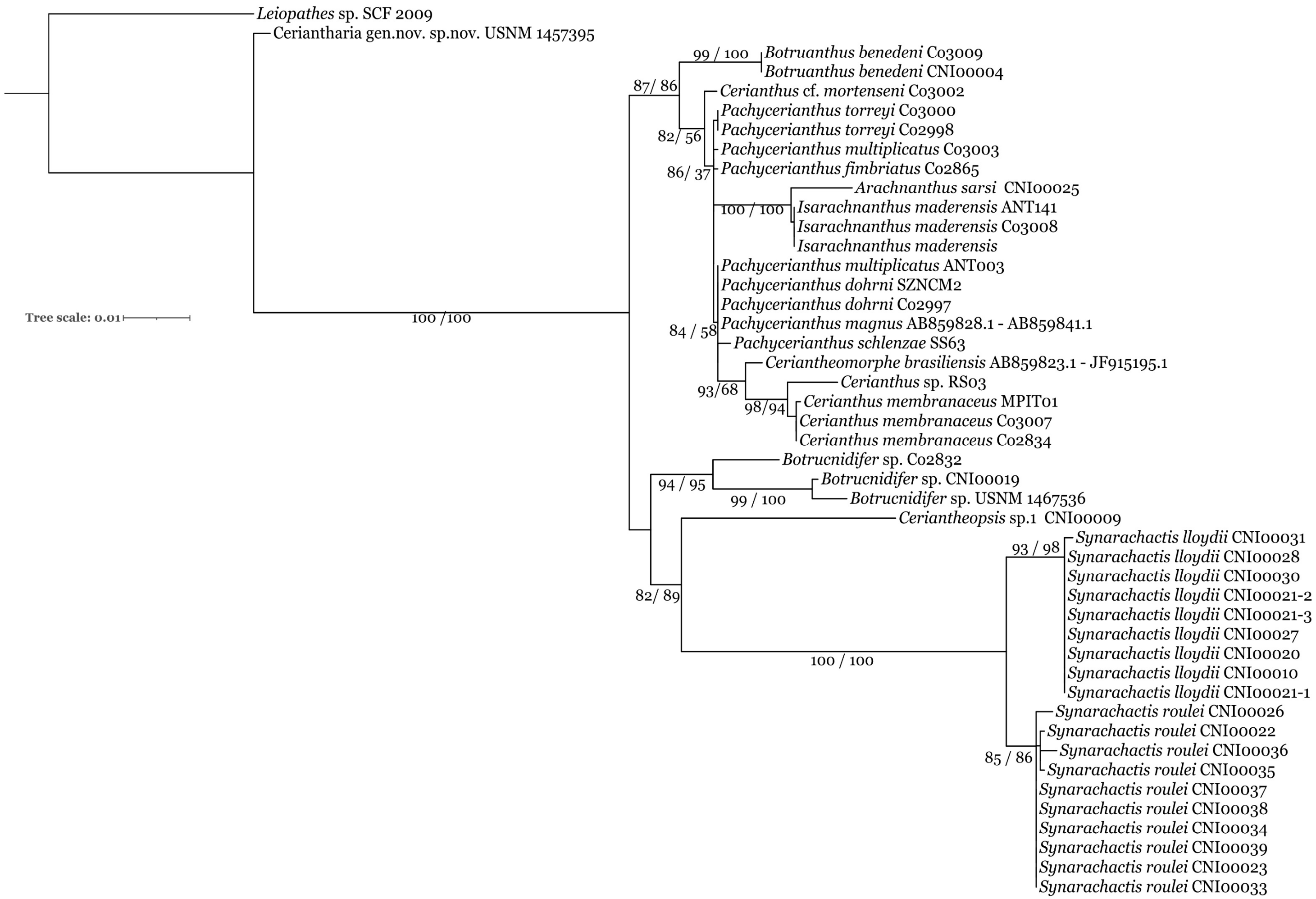

Supplementary Figure S5. 18S BI phylogenetic reconstruction. Posterior probabilities are shown. Model selected: SYM+I+G.

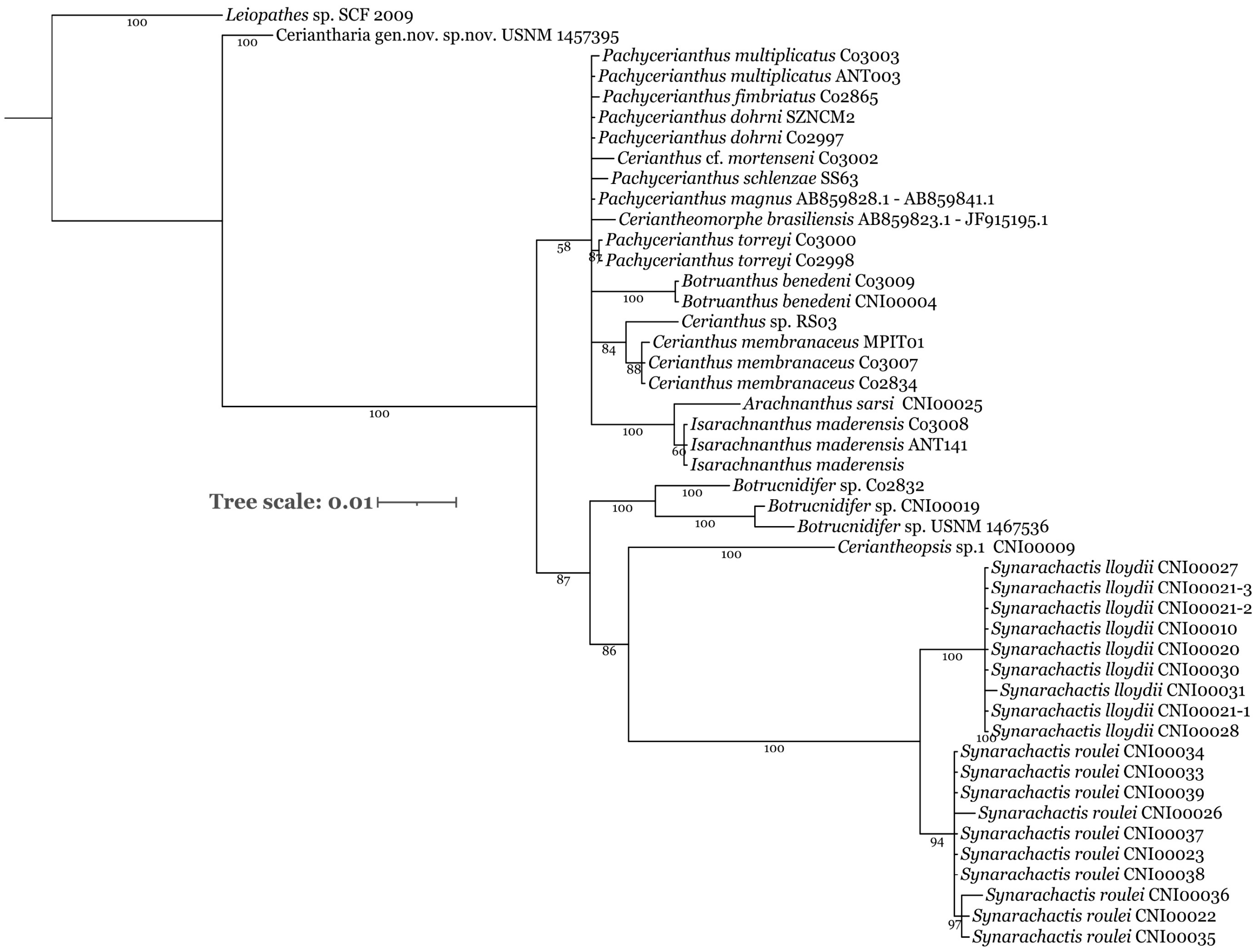

Supplementary Figure S6. Clade A COI BI phylogenetic reconstruction. Posterior probabilities are shown. Model selected: HKY: COI (1 position), K80: COI (2 position), F81: COI (3 position).

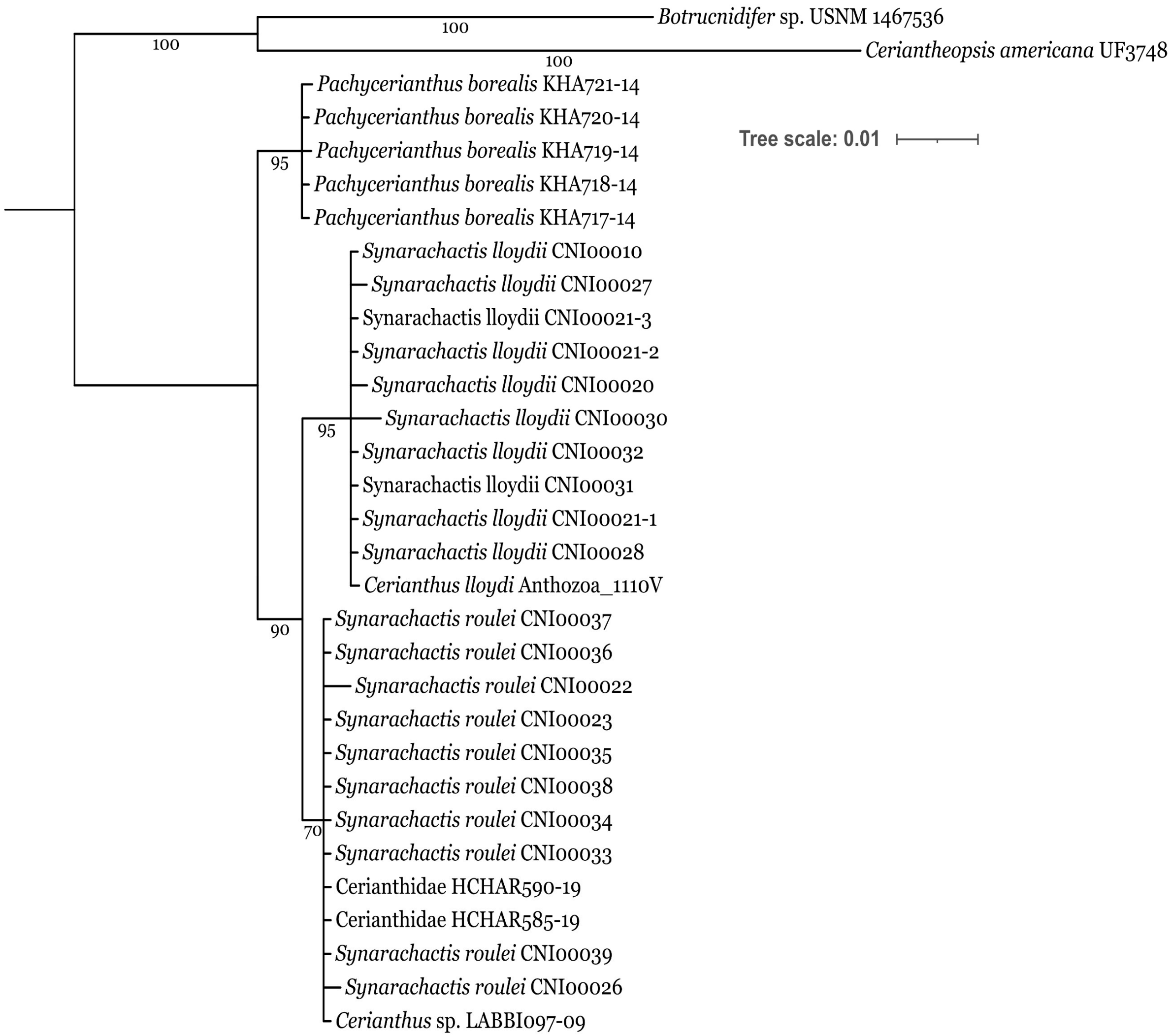

Supplement: Supplementary file 1 [file biology-12-01167-s001.zip › biology-2478519-supplementary.pdf]
